# Solid Contact Potentiometric Sensors Based on a New Class of Ionic Liquids on Thiacalixarene Platform

**DOI:** 10.3389/fchem.2018.00594

**Published:** 2018-11-27

**Authors:** Pavel L. Padnya, Anna V. Porfireva, Gennady A. Evtugyn, Ivan I. Stoikov

**Affiliations:** ^1^Department of Organic Chemistry, A. M. Butlerov' Chemistry Institute, Kazan Federal University, Kazan, Russia; ^2^Department of Analytical Chemistry, A. M. Butlerov' Chemistry Institute, Kazan Federal University, Kazan, Russia

**Keywords:** ionic liquid, solid-contact potentiometric sensor, phosphate determination, thiacalix[4]arene, carbon nanotubes

## Abstract

New solid-contact potentiometric sensors have been developed for hydrogen phosphate recognition on the basis of ionic liquids containing tetrasubstituted derivatives of thiacalix[4]arene in *cone* and *1,3-alternate* conformations with trimethyl- and triethylammonium fragments at the lower rim substituents. The recognition of selected anions including carbonate, hydrogen phosphate, perchlorate, oxalate, picrate, and EDTA was conducted using electrochemical impedance spectroscopy with ferricyanide redox probe. For the potentiometric sensor assembling, the ionic liquids were stabilized by multiwalled carbon nanotubes and carbon black deposited on the glassy carbon electrode. The influence of support, steric factors and modification conditions on the sensor performance has been investigated. As was shown, potentiometric sensors developed make it possible to selectively determine hydrogen phosphate anion within the concentration range from 1 × 10^−2^ to 1 × 10^−6^ M and limit of detection of 2 × 10^−7^−1 × 10^−6^ M with unbiased selectivity coefficients varied from 1.2 × 10^−1^ to 1.0 × 10^−8^ (carbonate, acetate, oxalate, succinate, glutharate, glycolate, and malonate anions).

## Introduction

Ionic liquids (ILs) are organic salts with the melting point below 100°C (Sun and Armstrong, 2010). Most of the ILs have a large cation (imidazolium, pyridinium, phosphonium, ammonium etc.) combined with rather small inorganic (Cl^−^, PF${}_{6}^{-}$, BF${}_{4}^{-}$) or organic (trifluoromethylsulfonate, trifluoroethanoate) anion. Relatively high difference in the ion size and their low symmetry result in rather low melting point of ILs. Besides, ILs offer some advantages of application in many areas, e.g., extraction, electrophoresis, liquid chromatography, and sensors. The ILs substitute conventional organic solvents in chemical synthesis and separation technologies. Due to extremely low volatility, thermal stability, non-flammability, low toxicity and reusability their application is considered as a green chemistry approach (Marr and Marr, 2016) though the synthesis of the ILs themselves utilizes some toxic species and reagents, which are not friendly for the environment. It is also important that many of the ILs features can be altered by design of the ions implemented in their content.

Regarding application of the ILs in electroanalytical chemistry (Sun and Armstrong, 2010; Tan et al., 2012) and in electrochemical sensors (Wei and Ivaska, 2009; Shiddiky and Torriero, 2011; Silvester, 2011), their undisputable advantages like ionic conductivity and chemical and electrochemical inertness are mostly mentioned. The addition of the ILs to the carbon paste (Švancara et al., 2009) or to the mixtures of carbonaceous materials (Abo-Hamad et al., 2016; Valentini et al., 2016) instead of paraffin improve the performance of biosensors due to lower intrinsic resistance of the electrode material. The synergic influence of ILs and redox active additives was reported for the voltammetric determination of oxidizable compounds (Sanati et al., 2017; Mohammadian et al., 2018). Better conditions of the electron transfer, enhanced sensitivity of the sensor reported due to IL introduction are attributed to the microextraction of the analyte molecules into the electrode body, its pre-concentration on the analysis stage (Nawała et al., 2018) and to microstructuring and self-gelation of polymeric layers on the transducer surface (Carvalho et al., 2014).

In potentiometric sensors, the ILs that are melted slightly above ambient temperature can be easily implemented in the electrode material as an ionophore or ion-exchanger (Shvedene et al., 2011). Their use allows excluding polymeric matrices and, in some cases, lipophilic salts from the surface membrane content. This is especially important for the development of solid-contact sensors where ILs serve as ion-to-electron materials required to establish reliable reversible response to primary ions. Although, analytical characteristics of such IL based potentiometric sensors are comparable with those of conventional ion-selective electrodes with internal filling, they are much easier *to prepare and store*, do not require time consuming conditioning prior to use and do not offer additional requirements in miniaturization and automation of potentiometric measurement systems.

The number of primary ions determined with ILs based sensors is increasing every year. To date, the determination of I^−^ (Shvedene et al., 2011; Mendecki et al., 2016), SO${}_{4}^{2-}$ (Peng et al., 2008), Cl^−^, I^−^, and SCN^−^ (Rzhevskaia et al., 2016), Cu^2+^ (Wardak and Lenik, 2013; Fan et al., 2017), Cd^2+^ (Afkhami et al., 2012; Wardak, 2015), Pb^2+^ (Wardak, 2011), Pr^3+^ (Ganjali et al., 2009), alkali metal cations (Xie et al., 2013). Besides, some organic species bearing charge or able to protonate/deprotonate have been determined with the IL based potentiometric sensors (triethylammonium-*closo*-dodecaborate; Kopytin et al., 2012; Faridbod and Shafaat, 2017). In some of them, ILs are used as binding material in rather complex matrix consisting of ionophores and redox active species. Thus, tramadol was determined with carbon paste sensor involving nanographene particles, tramadol-imprinted polymer and 1-butyl-1-methylpyrrolidinium bis (trifluoromethylsulfonyl)imide (Bagheri et al., 2017). The same IL was introduced in the carbon paste together with graphene nanosheets for determination of losartan (Bagheri et al., 2015). Electronic tongue system consisted of low-selective potentiometric sensor with 1,3-dihexadecylimidazolium salts as ionophores was successfully applied for identification of mineral water samples and separate determination of iodide and chloride anions in such waters (Shvedene et al., 2017).

Potentiometric sensors for anion determination are of a special concern due to limited number of ionophores because of the more complex requirements to their structure and binding sites against cation receptors. In the above examples of potentiometric sensors for anion detection, most of the ILs contain target anion to be detected in their structure as a counter ion. Meanwhile, the selectivity of their response remains moderate and the number of particular ions detected is rather limited. In some cases, variation of the lipophilic cation of the IL in a single sensor or assembling of the sensor array with different ILs were used to overcome this limitation.

Among inorganic anions, hydrogen phosphate anion is one of most frequently determined in the water and soil analysis as a part of mineral fertilizers and a marker of eutrophication and water contamination with stock farming wastes. Nevertheless, the number of ion-selective electrodes for hydrogen phosphate determination is rather limited. Insoluble salts and complexes of multivalent cations (Tonelli et al., 2013; Tafesse and Enemchukwu, 2015), oxyions (Wroblewski et al., 2000; Sessler et al., 2006), and polyamines (Carey and Riggan, 1994; Hartley et al., 2000) have been described in the assembly of appropriate sensors. Besides, molecular imprinting technology was recently proposed for the synthesis of polymeric membranes for phosphate recognition (Alizadeh and Atayi, 2018; Storer et al., 2018). The potentiometric sensors for HPO${}_{4}^{2-}$ determination show satisfactory characteristics but need to be improved in selectivity and sensitivity of target ion determination.

Calixarenes and their thia-analogs are macrocyclic compounds that offer unique opportunities of ionophore design due to the variety of functional groups in the substituents of the lower and upper rim of the macrocycle and pre-determined rigid spatial configuration of binding sites easily adapted to the guest molecule (ion) (Patra et al., 2012). The advantages of calixarene based receptors have been applied in the design of hydrogen phosphate receptors with good selectivity and sensitivity of the response (Yan et al., 2007). Calix[4]arene modifier was proposed for determination of inorganic anions including phosphates in snow water by capillary electrophoresis (Fernández-Gutiérrez et al., 2000). Indirect determination of organic acids by polyaniline based solid-contact sensors with functionalized thiacalix[4]arenes was described for general assessment of beverages (Evtugyn et al., 2010). Besides, thiacalix[4]arene based ionophores were used for discrimination of some inorganic anions and detection of ionic content of mineral waters (Sorvin et al., 2018).

In this work, we have combined the advantages of macrocyclic ionophores and ILs in the assemblies of solid-contact potentiometric sensors and developed potentiometric sensor to hydrogen phosphate with carbonaceous materials (carbon black (CB), multi-walled carbon nanotubes (MWCNTs)) as the IL supports. To the best of our knowledge, this is a first example of application of ILs with calixarene core for potentiometric determination of hydrogen phosphate.

## Materials and methods

### Reagents and materials

**Figure 1:**
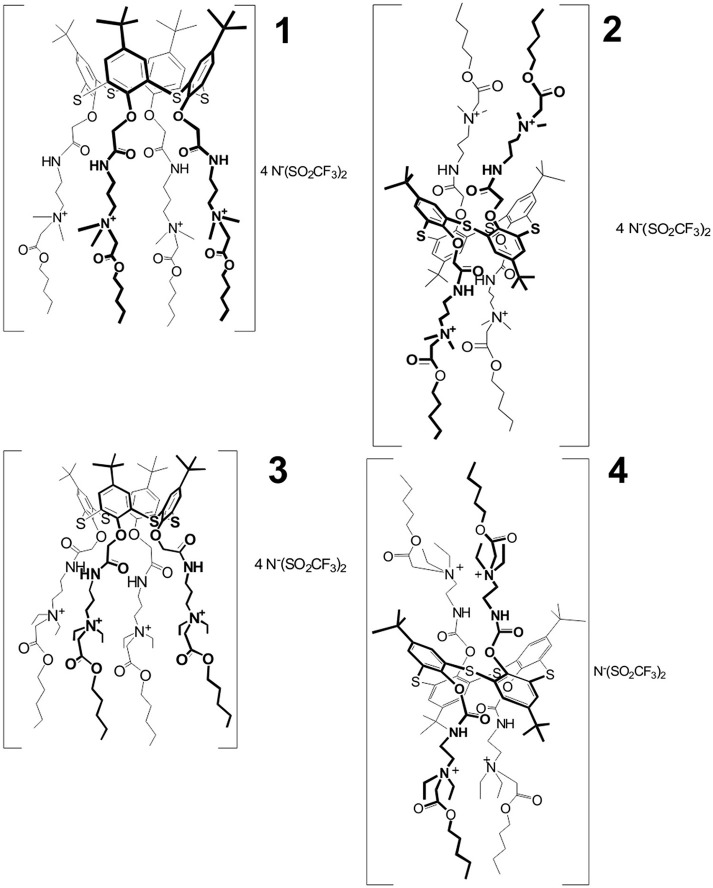
Chemical structures of the ILs used in the assembly of potentiometric sensors.

The ILs used in the work, i.e., 5,11,17,23-tetra-*tert*-butyl-25,26,27,28-tetrakis [(*N*-(3′,3′-dimethyl-3′-(pentoxycarbonylmethyl)ammoniumpropylcarbamoylmethoxy]-2,8,14,20-tetrathiacalix[4]arene tetra [bis(trifluoromethylsulfonyl)imide] in *cone* (**1**) and *1,3-alternate* (**2**) conformation, and 5,11,17,23- tetra-*tert*-butyl−25,26,27,28-tetrakis[(*N*-(2′,2′-diethyl-2′-(pentoxycarbonylmethyl) ammoniumethyl)carbamoylmethoxy]-2,8,14,20-tetrathiacalix[4]arene tetra[bis(trifluoromethylsulfonyl)imide] in *cone* (**3**) and *1,3-alternate* (**4**) conformation, were synthesized at the Organic Chemistry Department of Kazan Federal University as described elsewhere (Padnya et al., 2017). The conformation and chemical structure of the compounds were confirmed by ^1^H, ^13^C NMR, and IR spectroscopy, MALDI-TOF mass spectrometry and elemental analysis. Chemical structures of the IL studied are presented in Figure 1.

Oxalic acid, potassium ferricyanides K_3_[Fe(CN)_6_] and K_4_[Fe(CN)_6_], MWCNTs were purchased from Sigma-Aldrich, Germany, CB from IMERYS, Belgium, succinic acid from VitaChim, Russia, glutaric and glycolic acids from TauRus, Russia, malonic acid from AquaChim, Russia. All the reagents were of analytical grade. All the solutions were prepared using deionized Milli-Q® water.

Home-made glassy carbon electrode consisted of 2 cm long rod 1.7 mm in diameter inserted in the polytetrafluoroethylene tube was used for solid-contact ion-selective electrode assembling. Threaded connection made of stainless steel was fixed from the opposite end of the electrode. All the potentials are given against double-junction Ag/AgCl/3 M KCl reference electrode (Metrohm Autolab).

### Impedimetric measurements

EIS measurements were performed in working cell containing 4.5 mL of 0.1 M KNO_3_ and 0.5 mL of the mixture of 0.1 M K_3_[Fe(CN)_6_] and 0.1 M K_4_[Fe(CN)_6_] at 0.265 V using FRA2 module of the potentiostat-galvanostat PGSTAT 302N (Metrohm Autolab b.v., the Netherlands). The glassy carbon electrode was first mechanically polished and cleaned by acetone, sulfuric acid, NaOH and twice with deionized Milli-Q® water. Then, 2 μL of 1.0 mM solution of the IL in acetone were placed on the working surface and allowed to dry at ambient temperature. The amplitude of the applied sine potential was 5 mV and the frequency varied from 100 kHz to 0.04 Hz with a sampling rate of 12 points per decade. The capacitance and charge transfer resistance were calculated by fitting experimental data using Randles equivalent circuit (1) with the NOVA software (Metrohm Autolab).


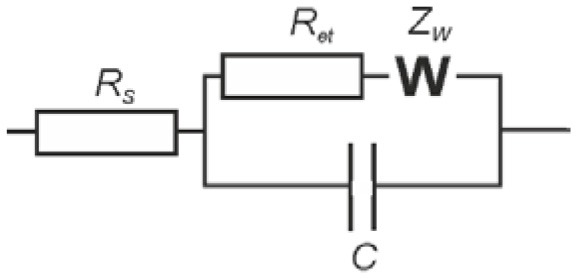


Here *R*_*s*_ is solution resistance, *R*_*et*_ charge transfer resistance, *Z*_*W*_ Warburg impedance and *C* is constant phase element which is here equal to interface capacitance.

Prior to measurement, freshly prepared electrode covered with the IL film was repeatedly heated to 80°C in solid-state thermostat and cooled to ambient temperature to equalize the surface layer. After equalization, an aliquot of the analyte solution was placed on the working surface for 10 min. Then the electrode was washed and the impedance was measured in the presence of the ferricyanide redox probe. The EIS parameters were calculated and averaged for three replications.

### Potentiometric sensor preparation and application

The glassy carbon electrode was first mechanically polished and cleaned as described above in section Impedimetric Measurements. In layer-by-layer deposition, 1 μL of 1 mg/mL CB suspension in dimethylformamide (DMF) was placed on its surface and dried at 60°C for 30 min. Then, 2 μL of 1.0 mM IL solution in acetone were placed on the electrode surface and dried again. Alternatively, a mixture of the CB suspension and IL solution in 10:1 or 20:1 v/v ratio was prepared and the same aliquot (2 μL) were spread on the working surface as described above.

MWCNTs were deposited on the surface of electrode in a similar manner. They were first mixed with concentrated H_2_SO_4_ and HNO_3_ in 3:1 vol. ratio and sonicated at 40°C for 4 h (Tesla ultrasonic bath, 40 W). Then they were centrifuged, washed with distilled water and dried at 80°C for 2 h. The MWCNTs dispersion was prepared by 15 min unltrasonication of oxidized MWCNTs in DMF.

SEM images of the CB and MWCNTs coatings were obtained with the high-resolution field emission scanning electron microscope Merlin™ (Carl Zeiss). Highly ordered pyrolytic graphite plate 12 × 12 × 2 mm (Agar Scientific, cat. No AG3389-1212) was used as substrate for deposition of carbonaceous materials.

If not used, the potentiometric sensors were stored in dry conditions in alumina foil cover at ambient temperature. Prior to use, dry electrodes were conditioned in deionized water and appropriate salt containing the anion to be determined.

Potentiometric measurements were performed at ambient temperature in 5 mL non-thermostated working cell with four-channel digital ionometer Expert-001 (Econix-Expert, Moscow, Russia). For sensor calibration, a series of standard solutions with concentration varied from 0.1 μM to 10.0 mM was prepared in deionized water. The pH was adjusted to 8.0 with NaOH. The pH of solutions was measured using glass pH-electrode (Econix-Expert). Calibration curves were obtained by step addition of appropriate salt solutions to the working cell under continuous magnetic stirring. The unbiased potentiometric selectivity coefficients ${}^{K_{i,j}^{Sel}}$ were determined by separate solution method (SSM). For this purpose, standard potentials of primary (${}^{E_{i}^{0}}$) and interfering (${}^{E_{j}^{0}}$) ions were calculated from the dependence of the electrode potential on their activities, *S*_*i*_ is the slope of calibration curve. The ${}^{K_{i,j}^{Sel}}$ values were calculated from Equation (2) adapted for anion determination (Bakker, 1997) and averaged from five replications.

(2) $$\begin{array}{r} \underline{\log K_{i,j}^{Sel}=-\frac{\left(E_{j}^{0}-E_{i}^{0}\right)}{S_{i}/z_{i}}} \end{array}$$

Besides, matched potential method (MPM) was used for selectivity coefficient determination to take into account non-ideal behavior of primary and interfering ions (Umezawa et al., 1995). In this method, changes in the potential are measured by addition of the primary ion to its reference solution. After that, interfering ion is added to the same reference solution to reach the same potential shift. The $K_{\text{i,j}}^{\text{Sel}}$ values are calculated in accordance with Equation (3), where $a_{\text{i}}^{0}$ is the activity of the primary ion in reference solution, *a*_*i*_ is its activity after addition of an aliquot, *a*_*j*_ is the activity of interfering ion resulted in the same shift of the potential.

(3) $$\begin{array}{r} \underline{K_{i,j}^{Sel}=-\frac{a_{i}-a_{i}^{0}}{a_{j}}} \end{array}$$

Contrary to SSM, MPM is insensitive to the difference in the charge of the primary and interfering ion and can be applied for non-Nernstian behavior of the ions.

The content of ionic species in the samples of lake waters was determined by ion chromatography using two-channel ICS-5000 system (Dionex, CA, United States) with CD conductivity detector.

## Results

### Impedimetric investigation of ionic liquids based on thiacalix[4]arene core

**Figure 2:**
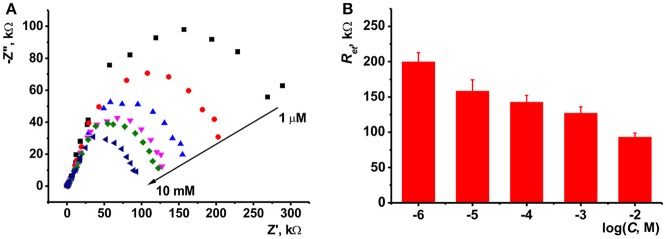
**(A)** The Nyquist diagram of impedance spectra recorded with glassy carbon electrode covered with IL **1** in the presence of 1.0 × 10^−2^, 1.0 × 10^−3^, 1.0 × 10^−4^, 1.0 × 10^−5^, and 1.0 × 10^−6^ M Na_2_HPO_4_. Measurements in the presence of 0.01 M K_3_[Fe(CN)_6_] and 0.01 M K_4_[Fe(CN)_6_] at 265 mV vs. Ag/AgCl. Frequency range 0.04 Hz−100 kHz, amplitude 5 mV. **(B)** The dependence of the charge transfer resistance on the concentration of hydrophosphate anion, mean ± S.D. for three replications.

Although, the ILs assume different mechanisms of potentiometric response toward target analytes, ion exchange remains most frequently discussed. To estimate the anion exchange capabilities of the ILs studied, EIS technique was used. In these experiments, the IL was deposited alone on the surface of glassy carbon electrode and the EIS spectra were recorded in the presence of ferricyanide redox probe. Reproducible results were obtained after equalization of the freshly prepared sensor in water. Commonly, in consecutive measurements the charge transfer resistance *R*_*et*_ first monotonously increased by 10–20 kΩ. In deionized water, the *R*_*et*_ was stabilized at about 200 kΩ. In the salts of inorganic and organic acids, stationary value was remarkably higher and depended on the analyte nature. Increase in the concentration of sodium phosphate, oxalate and carbonate in the range from 1.0 × 10^−6^ to 1.0 × 10^−2^ M decreased the *R*_*et*_. Between measurements, the potentiometric sensor was shortly heated above IL melting point (60–80°C) and sharply cooled to ambient temperature to accelerate the stabilization of the EIS parameters. Typical Nyquist diagram and changes in the charge transfer resistance are shown in Figure 2 for hydrogen phosphate and IL **1** as example. The slope of appropriate graphs in the plots of *R*_*et*_ vs. log*c* increased from 21 to 24 kΩ/pC (hydrogen phosphate and oxalate anions) to 65–70 kΩ/pC (carbonate and EDTA). Other anions tested influenced the *R*_*et*_ value only at their high concentrations. Thus, perchlorate suppressed the *R*_*et*_ value in concentrations exceeding 1.0 × 10^−3^ M, thiocyanate, chloride, bromide anions in those higher than 1.0 × 10^−2^ M.

Changes in the *R*_*et*_ values can be attributed to the ion exchange on the surface of the IL film. Partial substitution of bis(trifluoromethylsulfonyl)imide (Tf_2_N^−^) anion with a smaller ferricyanide ion bearing higher charge results in electrostatic repulsion of redox probe and hence increase of the *R*_*et*_ value. In the presence of other anions, the implementation of ferricyanide ions becomes lower and thus the resistance changes in the opposite direction. The lower the size of the anion and the higher its charge are the more the *R*_*et*_ value changes with its addition. The highest sensitivity corresponds to the three basic EDTA anion and carbonate. The latter one can additionally alter the accessibility of ferricyanide ion due to formation of molecular form CO_2_·H_2_O in the measurement conditions. All the anions mentioned above affect the *R*_*et*_ value in the whole range of concentrations tested, i.e., from 1.0 × 10^−6^ to 1.0 × 10^−2^ M.

Direct deposition of the ILs on the glassy carbon electrode allowed impedimetric detection of some anions able to ion exchange on the electrode interface. However, the lifetime of the sensor was limited by 15–20 measurements due to partial deterioration of the surface layer especially on the stage of heating and following solidification. This calls for searching possible supports for mechanical hardening of the surface layer and improving its robustness in routine measurements.

### Potentiometric sensors based on ionic liquids in the matrix of carbon black or carbon nanotubes

#### Choice of the supporting material

Carbonaceous materials, e.g., CB (Paczosa-Bator, 2014), graphene nanosheets (Afkhami et al., 2015; Shirzadmehr et al., 2015, 2016; Bagheri et al., 2016) and MWCNTs (Afkhami et al., 2014; Roy et al., 2017), are used in solid-contact sensors together with ionophores to improve the conditions of the signal transduction due to electron-to-ion conductivity of such materials. Besides, they can be applied as mechanical support of the liquid components preventing their leaching from the surface film.

We have used CB and MWCNTs deposited on the glassy carbon electrode prior to or together with the ILs. No lipophilic salts and plasticizers were necessary to establish a reliable reproducible response to the anions tested previously with EIS.

**Figure 3:**
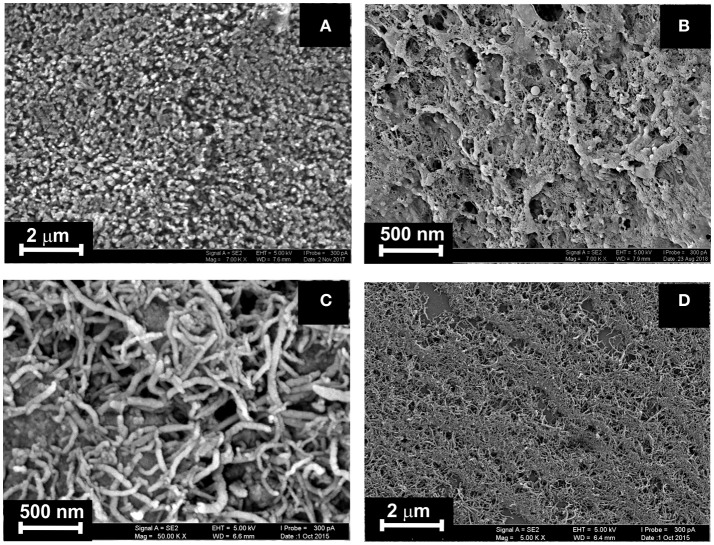
SEM images obtained for highly oriented pyrolytic graphite covered with CB **(A,B)** and MWCNTs **(C, D)** prior to **(A, C)** and after deposition of IL **1 (B, D)**. Layer-by-layer deposition, 0.1 mg/mL of CB and MWCNTs (aliquot 1 μL) + 0.1 mM IL **1** (aliquot 2 μL).

The ILs studied were deposited on the surface of electrode after formation of the underlying layer of the carbonaceous particles (layer-by-layer deposition) or in one step by preliminary mixing of the suspension of CB (MWCNTs) in DMF and IL solution in acetone. The amounts of CB and MWCNTs were chosen to reach full coverage of the electrode surface. Higher quantities of modifiers made worsen electron exchange conditions on the electrode interface and were less mechanically stable. In the series of consecutive drying-wetting steps, excessive amounts of particles left the electrode. This resulted in dramatic shift of the stationary potential of the sensor. As could be seen from Figure 3, the CB layer consisted of roundish particles 30–50 nm in size. Their coverage with the IL kept high porosity of the layer sufficient for a fast response. MWCNTs formed tangled three-dimensional layer with a high free internal volume then partially filled with the IL placed over.

The IL film improved the mechanical strength of the layer and about fully excluded deterioration of carbonaceous particles within the sensor lifetime. In some cases, crack appeared after washing the electrode, which could be removed by short heating them over 60°C and cooling to ambient temperature.

**Figure 4:**
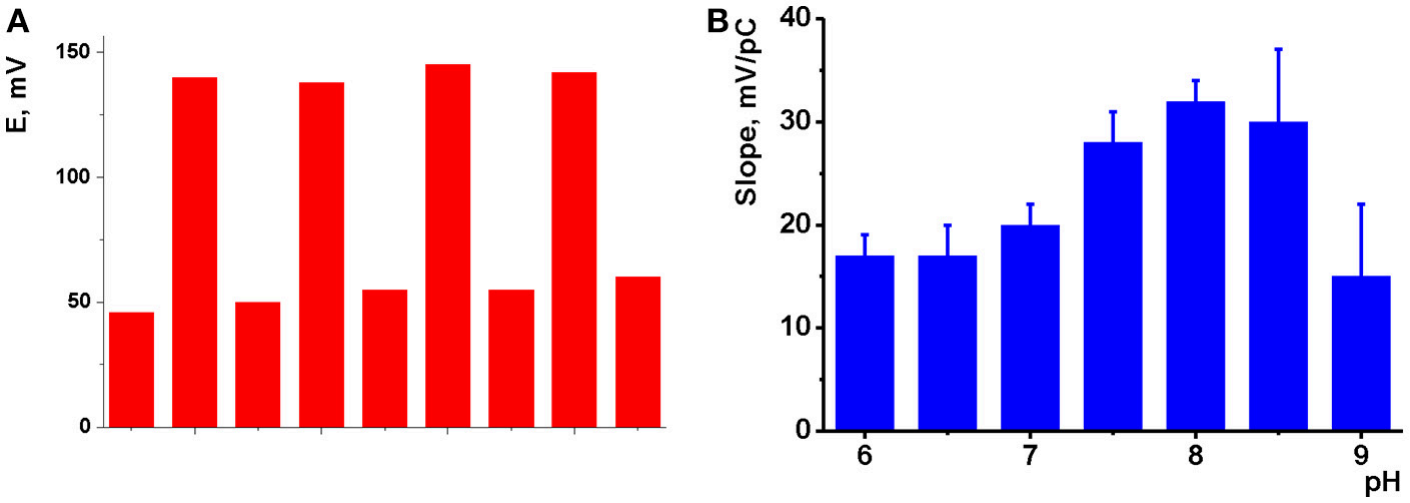
**(A)** Reversibility of the response of potentiometric to 10.0 mM and 10.0 μM Na_2_HPO_4_, pH 8.0, intermediate washing in deionized water and short heating to 80°C; **(B)** pH dependence of the slope of linear part of hydrogen phosphate calibration curve (mean ± S.D. for three replications). Potentiometric sensor based on sensor based on CB and IL **1**.

The potentiometric sensors were applied for the determination of Na_2_HPO_4_. Prior to use, freshly prepared sensor was conditioned overnight in 0.01 M solution of the analyte. The same results were obtained in series of heating—cooling steps as described above for impedimetric measurements. After addition of the hydrogen phosphate, the sensor potential shifts within 10–12 s in accordance with anionic sensitivity of the response. The dynamic response time insignificantly alters with the amounts of CB (MWCNTs) and IL present in the surface layer and tends to increase to 15–20 s with the analyte concentrations decreasing to 1.0 × 10^−6^ M. Reverse return after washing is slower but can be accelerated by melting IL in a short heating of the sensor to 80°C. The reversibility of the signal was demonstrated in measurements of alternating concentrations (10.0 mM and 10.0 μM) with the same sensor (Figure 4A).

#### pH influence

The pH value of the test solution is an important factor influencing the performance of potentiometric sensors (Afkhami et al., 2015; Shirzadmehr et al., 2015, 2016; Bagheri et al., 2016). To investigate the pH effect on the potentiometric response, first the potentials of glassy carbon electrode covered with CB or MWCNTs were measured at different pH values. The pH value was adjusted by addition of diluted nitric acid or sodium hydrochloride in the range from 2.0 to 10.0. A linear dependency was found with no respect of the direction of the pH change with the slope of −10 ± 2 mV/pH for CB and −8 ± 2 mV/pH for MWCNTs layer. The pH dependency was attributed to the carboxylic groups located on the surface of the particles of carbonaceous materials used. In basic media, deterioration of the surface layer took place after long incubation of the sensors. The addition of the ILs to the surface layer suppressed the slope of the pH dependency of the potential to 2–5 mV/pH, which is comparable with the standard deviation of the above parameter determined for six experimental points measured in triplicate. The influence of the ILs can be explained by shielding of the acidic groups on the surface of the coating and buffering influence of the anion of the IL that made the pH influence more smoothen against bare CB (MWCNTs) layer.

The response toward HPO${}_{4}^{2-}$ depended on the pH of solution to a much higher extent. The slopes of appropriate dependencies are presented in Figure 4B for CB based sensors as an example. One could see, they remain about constant at 16–18 mV/pC in neutral and weakly basic media, then increased to 30–32 mV/pH with maximum at pH 8.0 and then sharply decayed to 14–15 at higher pH values. The changes in the sensitivity of the response follow the distribution of phosphate ion forms and correspond to the domination of HPO${}_{4}^{2-}$ anions at pH > 8.0. The decrease of the slope value observed at higher pH was attributed to the mechanical deterioration of the film caused by electrostatic repulsion of negatively charged CB particles. In case of MWCNTs, maximal slope of the dependency remain constant in the pH range 8.0–10.0. However, the standard deviation of the slope increases to 10–14%. At pH < 8.0, dihydrogen phosphate becomes dominating and impedimetric measurements showed low sensitivity of the ILs to one-charge anions. Thus, the following measurements of the HPO${}_{4}^{2-}$ response were performed at pH 8.0.

#### Selection of the surface layer content

**Figure 5:**
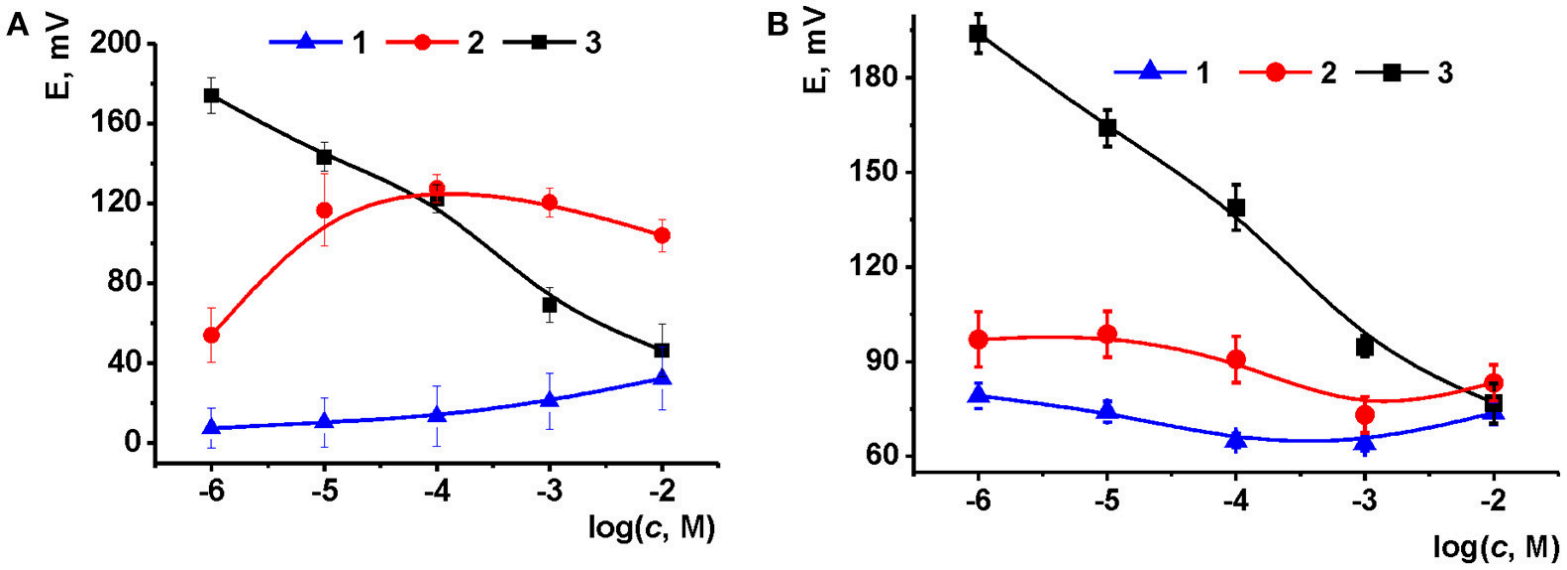
Calibration curves of Na_2_HPO_4_ obtained with the potential sensors based on CB **(A)** and MWCNTs **(B)** support (2 μL of 1.0 mg/mL suspension) and IL **1** [aliquot of 1 μL of 0.1 mM solution (1), 1 μL of 1.0 mM solution (2) and 2 μL of 1.0 mM solution (3)]. Layer-by-layer deposition, pH 8.0. Average from three replications (mean ± S.D. for three replications).

The content of the surface layer was specified by variation in the quantities of ILs added to the carbonaceous support (Figure 5). For CB based support, a weak cationic response was observed at low volume of IL solution deposited. This might be due to uncompensated anionic charge of carboxylate groups on the surface of the CB particles and electrostatic attraction of sodium cation from the solution. With increasing IL quantities, the calibration curve changed its direction and finally the slope of −33 mV/pC was reached typical from double charged ions. The following increase of the IL loading did not dramatically alter the position of the curve though the deviation of the potential became higher (up to 12%). The behavior of potentiometric sensors based on MWCNTs was similar: the maximal slope of the curve obtained for 2 μL aliquot of IL **1** solution was equal to 26 mV/pC. Thus, the following experiments were performed with 2 μL of 0.1 mM IL solution in acetone at pH 8.0.

#### Hydrophosphate ion determination

The analytical characteristics of hydrogen phosphate determination are presented in Tables 1, 2 for CB and MWCNTs based potentiometric sensors, respectively. All the standard solutions contained 0.1 M NaCl to take into account significant difference in their ionic strength. The comparison of the slopes showed higher results near Nernstian 29 mV/pC for the sensors obtained by layer-by-layer deposition of the components. Probably, adsorption of the ILs onto carbonaceous materials partially blocks the cationic centers involved in the interaction with the analyte ions. In case of CB/IL **1** loading, their simultaneous deposition in the mixture did not allow measuring reproducible response to HPO${}_{4}^{2-}$ anions. Variation in the content of the mixture did not significantly alter the analytical characteristics of the sensor. In case of IL **3**, super-Nernstian response was obtained in a narrow range of concentrations when mixture with CB was loaded on the electrode. This might be a result of non-stationary distribution of ions in the membrane body. Indeed, several cycles of heating-cooling of the sensor resulted in decrease of the slope to more convenient 25–27 mV/pC.

**Table 1:** Analytical characteristics of hydrogen phosphate determination with solid-contact potentiometric sensor based on CB and IL **1**-**4**.

**Surface layer content**	**E, mV** = **a** + **b**×**log(*****c*****, M)**			**Concentration range, M**	**LOD, M**
	**a**	**b**	***R*^2^**	
CB/**1** layer-by-layer	3.5 ± 1.2	−32.8 ± 1.3	0.9934	1.0 × 10^−6^−1.0 × 10^−2^	2.0 × 10^−7^
CB/**2** layer-by-layer	26 ± 1	−21.2 ± 0.3	0.9975	1.0 × 10^−6^−1.0 × 10^−3^	7.0 × 10^−7^
Mixture of CB/**2** 10:1	27 ± 2	−9.9 ± 1.6	0.9479	1.0 × 10^−6^−1.0 × 10^−4^	1.0 × 10^−6^
Mixture of CB/**2** 20:1	52 ± 5	−8.3 ± 1.1	0.9486	1.0 × 10^−6^−1.0 × 10^−3^	1.0 × 10^−6^
CB/**3** layer-by-layer	16.1 ± 1.4	−27.7 ± 0.3	0.9987	1.0 × 10^−6^−1.0 × 10^−3^	5.0 × 10^−7^
Mixture of CB/**3** 10:1	−290 ± 15	−73 ± 30	0.7216	1.0 × 10^−6^−1.0 × 10^−4^	1.0 × 10^−6^
Mixture of CB/**3** 20:1	−270 ± 25	−69 ± 9	0.8472	1.0 × 10^−6^−1.0 × 10^−4^	1.0 × 10^−6^
CB/**4** layer–by–layer	80 ± 9	−11.5 ± 2.1	0.9046	1.0 × 10^−6^−1.0 × 10^−3^	1.0 × 10^−6^
Mixture of CB/**4** 10:1	63 ± 22	−17.4 ± 4.4	0.8808	1.0 × 10^−6^−1.0 × 10^−4^	1.0 × 10^−6^
Mixture of CB/**4** 20:1	53 ± 32	−20.8 ± 6.3	0.8318	1.0 × 10^−6^−1.0 × 10^−4^	1.0 × 10^−6^

**Table 2:** Analytical characteristics of hydrogen phosphate determination with solid-contact potentiometric sensor based on MWCNTs and IL **1**-**4**.

**Surface layer content**	**E, mV** = **a** + **b**×**log(*****c*****, M)**			**Concentration range, M**	**LOD, M**
	**a**	**b**	***R*^2^**	
MWCNTs/**1** layer-by-layer	4.7 ± 3.1	−31.0 ± 1.9	0.9907	1.0 × 10^−6^−1.0 × 10^−2^	5.0 × 10^−7^
Mixture of MWCNTs/**1** 10:1	−16.2 ± 3.9	−7.4 ± 0.8	0.9785	1.0 × 10^−6^−1.0 × 10^−4^	1.0 × 10^−6^
Mixture of MWCNTs /**1** 20:1	−3.9 ± 3.7	−15.1 ± 0.7	0.9953	1.0 × 10^−6^−1.0 × 10^−4^	8.0 × 10^−7^
MWCNTs/**2** layer-by-layer	7.1 ± 1.9	−27.1 ± 1.2	0.9943	1.0 × 10^−6^−1.0 × 10^−4^	2.0 × 10^−7^
Mixture of MWCNTs/**2** 10:1	36 ± 3	−12.8 ± 2.7	0.9623	1.0 × 10^−5^−1.0 × 10^−3^	5.0 × 10^−6^
Mixture of MWCNTs /**2** 20:1	18.7 ± 3.4	−19.3 ± 1.2	0.9893	1.0 × 10^−6^−1.0 × 10^−3^	1.0 × 10^−6^
MWCNTs/**3** layer-by-layer	8.0 ± 2.8	−27.3 ± 1.8	0.9904	1.0 × 10^−6^−1.0 × 10^−2^	1.0 × 10^−6^
Mixture of MWCNTs/**3** 10:1	−74 ± 7	−18.8 ± 2.2	0.9745	1.0 × 10^−6^−1.0 × 10^−4^	1.0 × 10^−6^
Mixture of MWCNTs /**3** 20:1	−53 ± 6	−21.2 ± 5.0	0.9490	1.0 × 10^−6^−1.0 × 10^−3^	1.0 × 10^−6^
MWCNTs/**4** layer-by-layer	−15.0 ± 4.6	−17.8 ± 1.3	0.9781	1.0 × 10^−6^−1.0 × 10^−3^	1.0 × 10^−6^
Mixture of MWCNTs/**4** 10:1	−3.9 ± 1.0	−10.6 ± 0.7	0.9823	1.0 × 10^−6^−1.0 × 10^−2^	1.0 × 10^−6^
Mixture of MWCNTs /**4** 20:1	−35 ± 3	−21.1 ± 2.1	0.9517	1.0 × 10^−6^−1.0 × 10^−3^	1.0 × 10^−6^

**Figure 6:**
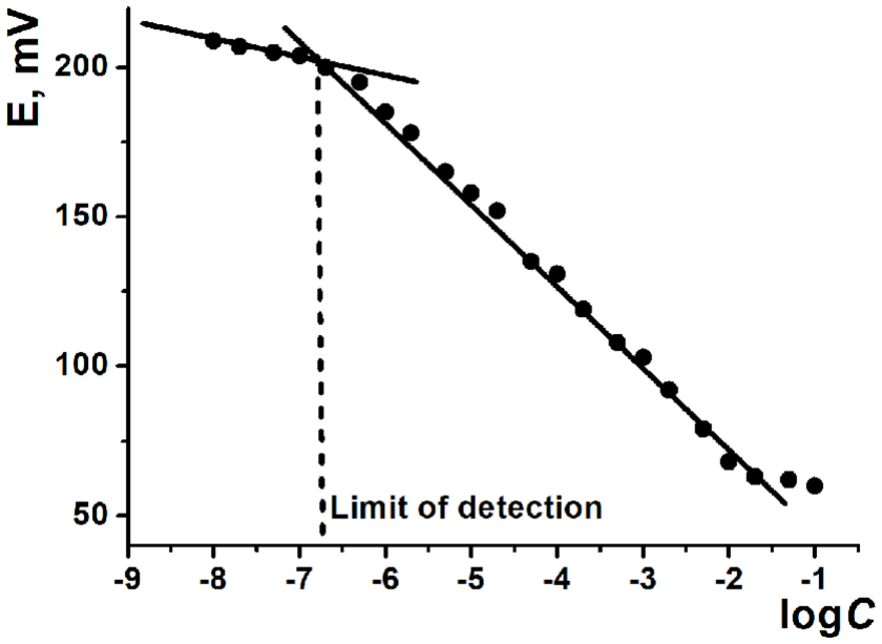
Determination of the limit of detection using calibration curve of Na_2_HPO_4_ determination with potentiometric sensor based on MWCNTs support and IL **1**.

The ILs based on thiacalix[4]arenes in *1,3-alternate* conformation are less sensitive to the deposition protocol but also demonstrate lower sensitivities of the HPO${}_{4}^{2-}$ determination against ILs on *cone* macrocycle platform. To some extent, the same refers to the difference between methyl- and ethylammonium derivatives (ILs **1**, **2** against **3**, **4**). In both cases, difference in the slopes of the curves can be attributed to different steric hindrance of the anion access to the cationic center of the IL. Contrary to that, CB and MWCNTs did not show significant difference in the hydrogen phosphate determination. Higher deviation of the signals observed for CB can be related to the lower reproducibility of the surface morphology and specific surface influencing the ion exchange. The LOD values were determined in accordance with the IUPAC recommendation (Buck and Linder, 1994) as interception point of the linear sections of the calibration plot (Figure 6).

The analytical characteristics of hydrogen phosphate determination are comparable or better than those reported for other potentiometric sensors (Table 3).

**Table 3:** Comparison of the analytical characteristics of HPO${}_{4}^{2-}$ ions determination with potentiometric sensors.

**Sensing layer**	**Dynamic range, M**	**LOD, M**	**Sample tested**	**References**.
Mo acetylacetonate complex in PVC matrix	1.0 × 10^−1^−1.0 × 10^−7^	6.0 × 10^−8^	Fertilizers, Ba^2+^ titration	Ganjali et al., 2006
Co/Co_3_(PO_4_)_2_	1.0 × 10^−1^−1.0 × 10^−5^	7.5 × 10^−6^	Microbial flocks	Lee et al., 2009
Tetraphenylazo macrocycle in PVC-polyurethane matrix	1.0 × 10^−1^−1.0 × 10^−6^	8.4 × 10^−7^	Standard reference material, titration	Kumar et al., 2010
Mg/Al hydrotalcite mineral in PVC matrix	5.0 × 10^−5^-2.0 × 10^−2^	3.0 × 10^−5^	Hydroxyapatite	Tonelli et al., 2013
AlPO_4_	1.0 × 10^−1^−1.0 × 10^−6^	1.0 × 10^−1^	-	Tafesse and Enemchukwu, 2015
Co phosphates	1.0 × 10^−1^−1.0 × 10^−5^	-	-	Hu et al., 2018
Polymer of methacrylate and vinyl pyridine with molecular imprints	1.0 × 10^−1^−1.0 × 10^−5^	4.0 × 10^−6^	La^3+^ titration	Alizadeh and Atayi, 2018
CB (MWCNTs) and IL with cations on thiacalixarene platform	1.0 × 10^−2^−1.0 × 10^−6^	(0.2–1.0) × 10^−6^	Lake water	This work

#### Measurement precision, sensor lifetime, and selectivity

The measurement precision toward 1.0 × 10^−4^ M HPO${}_{4}^{2-}$ ions was estimated using five individual sensors made with MWCNTs support from the same reagent set. Each sensor was used for five consecutive measurements of the hydrophosphate signal performed at pH 8.0 in the presence of 0.1 M NaCl. The measurement-to-measurement repeatability was equal to 1.5% and sensor-to-sensor repeatability to 2.5%. The lifetime of the sensor stored in dry conditions was assessed by every weak signal measurement. After 3 months, the signal toward 1.0 × 10^−4^ M HPO${}_{4}^{2-}$ ions decreased to 90% of its initial value. Thus, potentiometric sensor developed showed satisfactory stability and signal measurement precision convenient for real sample analysis. Similar experiments with the CB layer showed lower stability of sensing layer and life time of 6 weeks. Measurement-to-measurement and sensor-to-sensor repeatability were found to be 3.5 and 4.5%, respectively.

Using this potentiometric sensor, unbiased selectivity coefficients were determined by SSM and MPM methods (see section Potentiometric Sensor Preparation and Application). The results obtained are presented in Table 4. Except carbonate ion, most of the potentiometric sensors exhibited highly selective response to target analyte. MPM calculations result in higher selectivity coefficients especially in case of one-charged ions. This is a result of non-Nernstian behavior of interfering ions and incomplete consideration of different ion charges. In some cases with high difference in the signals of primary and interfering ions, the increment in the potential established for primary ion cannot be reached by addition of interfering ion. In such cases, dash sign in placed in brackets. The IL **3** can be considered as most selective receptor among other ILs studied. Selectivity of the solid-contact sensors developed makes it possible to determine hydrogen phosphate anions in natural and mineral water on the level of their permissible levels (5.0 × 10^−6^ M in Russian Federation).

**Table 4:** Potentiometric selectivity coefficients ${}^{K_{Phos,j}^{Sel}}$ of solid-contact potentiometric sensors based on MWCNTs and ILs **1**-**4** calculated by SSM and MPM methods.

**Interference**	**Potentiometric selectivity coefficients**${}^{K_{Phos,j}^{Sel}}$ **(SSM / PMP)**			
	**1**	**2**	**3**	**4**
Carbonate	1.2 × 10^−1^ (2.5)	2.9 × 10^−2^ (0.3)	1.7 × 10^−4^ (4.4 × 10^−2^)	4.8 / 6.0 (2.5)
Chloride	5.2 × 10^−9^ (-)	1.0 × 10^−6^ (5.0 × 10^−3^)	1.0 × 10^−7^ (8.2 × 10^−5^)	2.8 × 10^−6^ (4.5 × 10^−3^)
Thiocyanate	4.2 × 10^−7^ (5.0 × 10^−5^)	4.9 × 10^−5^ (2.0 × 10^−3^)	1.5 × 10^−8^ (-)	1.1 × 10^−5^ (4.2 × 10^−3^)
Bromide	9.0 × 10^−7^ (2.0 × 10^−5^)	8.4 × 10^−6^ (1.0 × 10^−4^)	3.2 × 10^−7^ (5.8 × 10^−5^)	2.4 × 10^−6^ (6.1 × 10^−5^)
Acetate	1.2 × 10^−8^ (-)	1.4 × 10^−5^ (5.5 × 10^−3^)	1.0 × 10^−8^ (-)	7.8 × 10^−4^ (4.3 × 10^−2^)
Oxalate	2.8 × 10^−7^ (-)	3.2 × 10^−5^ (6.7 × 10^−4^)	4.8 × 10^−6^ (6.4 × 10^−5^)	1.9 × 10^−5^ (8.3 × 10^−5^)
Succinate	7.6 × 10^−6^ (1.5 × 10^−4^)	1.4 × 10^−4^ (1.5 × 10^−3^)	3.1 × 10^−5^ (4.7 × 10^−4^)	5.9 × 10^−5^ (3.9 × 10^−4^)
Glutarate	4.6 × 10^−5^ (3.0 × 10^−3^)	1.7 × 10^−4^ (1.2 × 10^−3^)	1.7 × 10^−5^ (7.2 × 10^−3^)	3.4 × 10^−5^ (2.0 × 10^−4^)
Glycolate	8.7 × 10^−6^ (6.0 × 10^−4^)	1.5 × 10^−4^ (6.5 × 10^−4^)	2.1 × 10^−5^ (9.0 × 10^−4^)	6.6 × 10^−5^ (8.6 × 10^−4^)
Malonate	6.1 × 10^−6^ (2.0 × 10^−3^)	1.2 × 10^−4^ (6.5 × 10^−4^)	1.1 × 10^−5^ (7.1 × 10^−4^)	2.2 × 10^−5^ (6.1 × 10^−4^)

*‘-’, means that MPM method is not applicable*.

### Real sample analysis

The potentiometric sensors developed were tested in the determination of hydrogen phosphate anions in fresh waters samples taken from natural lakes in the suburb of Kazan City. The content of anions established by ionic chromatography and the results of potentiometric determination are presented in Table 5. CB/IL **1** was used in all the measurements, the results were averaged for three replications. As could be seen, the potentiometric sensor developed can be used for the assessment of the phosphate content in fresh waters. Minor underestimation against chromatography results can be attributed to the different sample treatment protocol including some other phosphate sources (organic esters, pyrophosphates) undetectable with potentiometric sensors. In potentiometric experiment, water samples were filtered to exclude organic phosphate sources and their pH was adjusted to 8.0 with NaOH solution.

**Table 5:** Phosphate determination in lake water samples with the potentiometric sensor based on CB/IL **1**.

**Anion**	**HCO_3_^−^ mg/L**	**Cl^−^, mg/L**	**NO_3_^−,^mg/L**	**NO_2_^−^ mg/L**	**PO**_**4**_ **(total), mg/L**	
					**Ion chromatography**	**Potentiometry (HPO${}_{4}^{2}$^−^ content)**
Sample 1	80.5	1.2	4.0	0.15	0.1	0.07 ± 0.02
Sample 2	45.0	2.2	12.0	0.35	0.6	0.5 ± 0.1
Sample 3	50.0	1.5	25.0	0.8	0.8	0.6 ± 0.1

*Other anions content was determined by ion chromatography*.

## Discussion

Application of ILs in the assembly of solid-state potentiometric sensors offers some attractive advantages, e.g., small size, flexibility of the design, no need in internal filling and long conditions prior to use. Besides, the ILs do not need in additional implementation of lipophilic salt and plasticizers and are applicable with modern technologies of sensor manufacture (screen-printing technique). Meanwhile, the number of successful examples of IL based potentiometric sensors is rather limited due to difficulties in their synthesis and some difficulties of introduction in the sensor assembly including moderate solubility in water and in some cases insufficient mechanical durability of the surface layer.

In this work, we propose to use a new class of ILs which is based on thiacalix[4]arene platform with rather long hydrophobic substituents bearing cationic centers able to exchange. Previously we have shown that introduction of Tf_2_N^−^ anion instead of bromide or nitrate decreased melting point to meet the IL definition (Padnya et al., 2017). Insolubility of the ILs tested in water is another advantage providing a long lifetime of the sensors and low potential drift during the operational period.

Direct EIS investigation of the films obtained by deposition of the ILs on electrode showed remarkable dependence of the charge transfer resistance to the anions present in the solution which was referred to the competition between the ferricyanide ions (redox probe) and those added to the solution for the binding sites of the IL layer. The comparison of the effect achieved for different salts made it possible to preliminarily conclude on the selective recognition of hydrogen phosphate anions. The following experiments with carbonaceous materials as supports for ILs confirmed the possibility for reliable and sensitive determination of HPO${}_{4}^{2-}$ anions in aqueous solutions. Ion exchange mechanism of potentiometric response toward HPO${}_{4}^{2-}$ anions was confirmed by EIS data. The comparison of the results obtained with *cone* and *1,3-alternate* conformations of macrocycle core as well as those with dimethyl- and diethyl-ammonium fragments in the substituents at the lower rim of the macrocycle confirmed the suggestion about predominant influence of steric factors on the recognition of the hydrogen phosphate anion. The conditioning of the freshly prepared sensor as well as its recovery after the contact with the sample can be accelerated by short heating to 80°C followed by solidification by cooling to ambient temperature. The sensor showed good mechanical durability and stable response toward 1.0 × 10^−2^−1.0 × 10^−6^ M HPO${}_{4}^{2-}$ within at least 30 days with a standard deviation varied from 2.3% (MWCNTs) to 3.2% (CB) in the series of six individual sensors. The selectivity of the response makes it possible to directly determine the hydrogen phosphate anion in the presence of most inorganic and many organic anions commonly present in water samples and biological fluids. The potentiometric sensor was validated on real samples of surface waters containing some contaminants typical for rural area (high quantities of nitrite and nitrate salts and moderate mineralization level). The results obtained can be used for further miniaturization of the sensor and extension of its possible area of application to biological fluids and wastes containing biogenic and inorganic phosphate sources.

## Author contributions

PP and IS synthesized ILs based on thiacalixarene platform, characterized their purity and conformation and participated in the manuscript preparation. AP and GE performed electrochemical measurements (EIS and potentiometry).

### Conflict of interest statement

The authors declare that the research was conducted in the absence of any commercial or financial relationships that could be construed as a potential conflict of interest.
